# Facing the fear of failure: An explorative qualitative study of client experiences in a mindfulness-based stress reduction program for university students with academic evaluation anxiety

**DOI:** 10.3402/qhw.v10.27990

**Published:** 2015-08-20

**Authors:** Aslak Hjeltnes, Per-Einar Binder, Christian Moltu, Ingrid Dundas

**Affiliations:** 1 Department of Clinical Psychology, Faculty of Psychology, University of Bergen, Bergen, Norway; 2 Department of Psychiatry, Hospital of Førde, Førde, Norway

**Keywords:** Mindfulness-based stress reduction, university students, college students, test anxiety, performance anxiety, evaluation anxiety, qualitative research, reflexivity, thematic analysis, phenomenology, hermeneutics

## Abstract

The aim of this qualitative study was to investigate the subjective experiences of 29 university students who participated in an 8-week mindfulness-based stress reduction (MBSR) program for academic evaluation anxiety. Participants who self-referred to the Student Counseling Service underwent individual semi-structured interviews about how they experienced the personal relevance and practical usefulness of taking the MBSR program. Interviews were transcribed and analyzed through a team-based explorative–reflective thematic approach based on a hermeneutic-phenomenological epistemology. Five salient patterns of meaning (themes) were found: (1) finding an inner source of calm, (2) sharing a human struggle, (3) staying focused in learning situations, (4) moving from fear to curiosity in academic learning, and (5) feeling more self-acceptance when facing difficult situations. We contextualize these findings in relation to existing research, discuss our own process of reflexivity, highlight important limitations of this study, and suggest possible implications for future research.

University students report a high prevalence of mental health problems, and research indicates a general increase in symptoms of mental disorders among students in higher education (Bayram & Bilgel, 2008; Eisenberg, Gollust, Golberstein, & Hefner, 2007; Storrie, Ahern, & Tuckett, 2010). Academic stress represents an important psychosocial challenge for both undergraduate and graduate students (Misra & McKean, 2000; Robotham & Julian, 2006). The high prevalence of mental health problems among university students highlights a need for accessible interventions that can address the specific psychosocial challenges of this population (Regehr, Glancy, & Pitts, 2013). Mindfulness-based interventions emphasize awareness, non-striving, and acceptance of present-moment experience, which may seem paradoxical for young people in a stressful academic situation, where achievement and high performance on exams may appear imminently important for their future life. How do university students experience the usefulness of participating in an 8-week mindfulness-based training program for severe academic evaluation anxiety? The present study aimed to explore the subjective experiences of 29 university students who participated in an 8-week mindfulness-based stress reduction (MBSR) program for academic evaluation anxiety.

The terms evaluation anxiety, academic performance anxiety, test anxiety, and exam anxiety refer to an anxiety in academic situations that may have severe consequences for well-being and academic learning. Performance anxiety is defined by DSM-V as a subtype of social anxiety disorder (SAD) (APA, 2013). Powell (2004) describes performance anxiety as “strong but delimited fears that severely compromise an individual's capacity to execute a task at a level that can be reasonably expected, which is crucial to that person's normal adjustment” (p. 804). The prevalence of academic evaluation anxiety has been estimated to be as high as 35% among college students, and has been shown to be associated with lower actual academic performance (Cassady & Johnson, 2002; Naveh-Benjamin, McKeachie, Lin, & Lavi, 1997). The potential negative consequences of academic performance anxiety during the college or graduate years indicate a need for accessible psychological interventions that may address the concerns of university students who experience these academic and emotional difficulties.

Mindfulness has been defined as a process of “paying attention, on purpose, in the present moment, and nonjudgmentally to the unfolding of experience moment by moment” (Kabat-Zinn, 2003, p. 145). The MBSR program is an 8-week course for groups who meet weekly for 2–3 h for instruction and practice in mindfulness meditation (Baer, 2003; Kabat-Zinn, 1990; Santorelli, 1999). The program involves systematic training in mindfulness exercises, Hatha yoga, group discussions, and homework between classes. The psycho-educational components emphasize management of stress reactions and difficult situations through awareness and acceptance of physical or psychological experience. MBSR is reported to be an effective intervention strategy for a broad spectrum of physical and psychological symptoms (Baer, 2003; De Vibe, Bjørndal, Tipton, Hammerstrøm, & Kowalski, 2012; Hofmann, Sawyer, Witt, & Oh, 2010). Randomized controlled trials (RCTs) have demonstrated the effectiveness of MBSR for people with different anxiety disorders (Kabat-Zinn et al., 1992; Vollestad, Nielsen, & Nielsen, 2012; Vollestad, Sivertsen, & Nielsen, 2011) and there are two recent RCTs that have reported MBSR as an effective intervention for SAD (Jazaieri, Goldin, Werner, Ziv, & Gross, 2012; Koszycki, Benger, Shlik, & Bradwejn, 2007). There is less empirical research on the impact of mindfulness-based interventions on individual performance, and although three recent studies indicate that mindfulness may facilitate improved performance in academic contexts (Beauchemin, Hutchins, & Patterson, 2008; Howell & Buro, 2011; Shao & Skarlicki, 2009), there is a need for empirical studies to examine the mindfulness-based interventions for university students with academic evaluation anxiety.

The present article is based on data from a larger clinical study of MBSR for university students with academic evaluation anxiety (which was conducted at University of Bergen, Norway). In a prior article (Dundas, Thorsheim, Hjeltnes, & Binder, 2015), we described the results of a naturalistic pilot study of the effectiveness of MBSR for university students with academic evaluation anxiety, finding reduced academic evaluation anxiety and improved self-confidence after the intervention. In the current article, the aim is to explore the subjective experiences of the participants before, during, and after the intervention.

What do clients with anxiety disorders actually experience when they participate in mindfulness-based group interventions, and how do they describe their personal experiences of suffering and therapeutic change? Elliott (2010) argues for the importance of explorative qualitative research in building direct knowledge of the processes of therapeutic change. This argument is parallel to the broader recognition in the field of psychosocial intervention research that well-performed qualitative studies are needed to enhance the clinical usefulness, conceptual robustness, and ecological validity of the knowledge base (Castonguay & Beutler, 2006; Malterud, 2001; McLeod, 2013). Qualitative studies of *how* psychological treatments are experienced by clients, and *what* clients themselves experience as the helpful or not helpful aspects of these interventions, could generate hypotheses for future research (Elliott, 2010; Mace, 2008). Specifically, because the majority of contemporary mindfulness research has been conducted within quantitative and neurobiological research paradigms, a continued examination of the first-person perspective and potential process variables is needed to understand how mindfulness-based interventions work for clients in specific contexts (Baer, 2010b; Kuyken et al., 2010; Shapiro, Carlson, Astin, & Freedman, 2006; Wyatt, Harper, & Weatherhead, 2014). Mace (2008) argues that there is a need for more qualitative studies in the emerging scientific literature on mindfulness, and that systematic qualitative methods may be used to “understand the needs of, and likely impacts on, specific client groups when undertaking interventions like the MBSR program” (p. 38). Two meta-syntheses conducted by Malpass et al. (2012) and Wyatt et al. (2014) have examined the existing qualitative research on mindfulness-based interventions, and indicate an absence of prior qualitative studies of client experiences in MBSR for anxiety disorders. To our knowledge, this is the first qualitative study of client experiences in mindfulness-based interventions for anxiety disorders. The present article attempts to address these gaps in the scientific literature by addressing the following exploratory research question: How do university students describe their experiences of taking the MBSR program to find ways of working with academic evaluation anxiety and other challenges in their everyday life?

## Method

### Methodological approach

To investigate the experiences of the university students taking the MBSR program, we chose an explorative–reflective thematic analysis methodology that has been used previously to study client and therapist experiences in psychotherapy (Binder, Holgersen, & Moltu, 2012; Braun & Clarke, 2006; McLeod, 2013). This methodological approach uses individual semi-structured interviews, and aims to provide experience-near and ecologically valid descriptions of complex psychological phenomena by combining phenomenological investigation of human experience with systematic interpretation of meaning through thematic analysis. The methodology follows three basic epistemic principles: (1) the research inquiry seeks to explore the phenomenological dimension of human lived experience, with a recognition that the “bracketing” of all prior assumptions by the research team can only be partially achieved, (2) meaning is in fundamental ways *co-created* in the encounter between interviewer and interviewee, and between the researcher and the data in subsequent analyses, and (3) the process of developing knowledge from the research participants’ descriptions of their experiences necessarily involves acts of interpretation from the researchers’ perspective (Adams & Leary, 2007). The hermeneutic-phenomenological framework of this research methodology emphasizes reflexivity and transparency in the research process (Alveson & Sköldberg, 2009; Finlay & Gough, 2003).

### Participants

The participants were recruited through announcements at the Student Welfare Center and through advertisements on campus. As they signed up for the MBSR course, students were informed about the research project so that they could give their informed consent, and underwent an initial screening. Exclusion criteria were: (1) active psychosis, (2) unstabilized bipolarity, (3) severe personality disorders (4) substance abuse/dependence, (5) flashbacks after trauma, and (6) severe self-harm or suicidality. No students were excluded due to these criteria. Seventy students (60 women and 10 men) were assessed to be eligible for the study.

Five MBSR courses were held over a period of one and a half years. The 8-week MBSR courses were based on the standard protocol for MBSR, involving 2.5 h of weekly sessions, daily homework assignments, and a 1-day retreat in the sixth week of the course (Santorelli & Kabat-Zinn, 2009). The psycho-educational components of the group discussions were adapted to the specific stressors and performance anxiety problems experienced by the university students, while maintaining fidelity to the structure and principles of the MBSR curriculum guide (Dobkin, Hickman, & Monshat, 2013). Fifteen students of the original 70 students dropped out during the course, whilst 55 students completed the course. Within 1 month after their first scheduled exam following completion of the MBSR course, we contacted participants in order to schedule an interview about their experiences with MBSR and their recent exam. Only one student asked not to be contacted for an interview. We continued interviewing participants until the research team evaluated the content of the conducted interviews and agreed that data saturation had been more or less achieved. The final sample consisted of 29 university students (25 females and 4 males), with a mean age of 28 years (median: 24 years, *SD=*8). The high average age reflects that the age-variable in the sample of students seeking help for academic evaluation anxiety was skewed toward younger participants. The interviewed sample included five students over 35 years of age, perhaps reflecting that evaluation anxiety may obstruct some students from completing their studies. At the time of participation, the participants were enrolled in bachelor and master programs in psychology, medicine, law, computer science, media, performing arts, and natural sciences.

### Researchers

The study was conducted within University of Bergen, Norway. The first, second, and fourth authors, who have taken professional training in MBSR at the Center for Mindfulness, acted as teachers in the clinical study. The third author does not have a background in mindfulness, and therefore acted as a critical auditor in the research group.

### Data collection method

Individual semi-structured in-depth interviews were conducted individually with the participants within 1 month of completion of their MBSR course, to facilitate an open exploration of what they had experienced during and after the MBSR program (Binder, Holgersen, & Moltu, 2012; Kvale & Brinkmann, 2009), while at the same time seeking answers to specific questions by including probes (for the interview guide, see Appendix). The aim was to increase the validity of our understandings during the interview by asking for concrete examples whenever possible, and “reflecting back” our understandings to the interviewee for him or her to correct. The aim of the interviews was to explore the experiences that the participants themselves held as important, while at the same time looking for descriptions of helpful and not helpful aspects of taking the MBSR program in as much concrete detail as possible. The interviews were conducted by the second, third, and fourth authors and a colleague at the Student Welfare Center. In no instances were interviews conducted by the instructor of the specific MBSR course that the student had attended. The interviews were recorded and transcribed verbatim for analysis.

### Data analysis

The data analyses of the transcribed interviews were conducted using a team-based explorative–reflective thematic analysis (Binder, Holgersen, & Moltu, 2012) and Nvivo 10 software (QSR, 2012). By comparing the individual accounts, we sought to identify patterns of meaning in how the university students experienced the MBSR program and the process of finding ways to master academic evaluation anxiety. Analysis proceeded through eight stages detailed in Table I.

**Table I:** Stages in the explorative–reflective thematic analysis.

Research principles	Description of the research process
**1**. Noting initial impressions:	The interviewers wrote down their immediate impressions after their dialogs with the participants. They discussed these observations to establish a basic sense of the participants’ experiences, and to promote reflexive awareness of interpersonal processes in the interview situation.
**2**. Familiarization with the data:	All researchers read the transcribed material to obtain a basic sense of the experiences described by the participants.
**3**. Searching for patterns of meaning:	Examining those parts of the text relevant to the research questions, the first and second author identified separable content units that represented different aspects of the participants’ experiences. We would here look at how the university students described their experiences of taking the mindfulness-based stress reduction (MBSR) program in relation to mastering academic evaluation anxiety and other challenges in their everyday life.
**4**. Coding themes:	The first and second author developed “meaning codes” for those units, which are concepts or keywords attached to a text segment in order to permit its later retrieval. The first author then edited the text in accordance with those codes into coded groups of text with the technical assistance of Nvivo 10 software. For example, the participants’ descriptions of being friendlier with themselves or tolerating their anxiety were given the code “Acceptance.”
**5**. Reviewing and summarizing themes:	The first and second author interpreted and summarized the meaning within each of the coded groups of text fragments into conceptions and overall descriptions of meaning patterns and themes reflecting what, according to their understanding, emerged as the most important aspects of the participants’ experiences.
**6**. Team-based revision of themes:	All four authors turned back to the overall text to check whether voices and points of view needed to be added, or whether the conceptions and descriptions of themes could be developed further.
**7**. Critical auditing:	The third author, who was not a part of the team of teachers in the MBSR groups, had a leading role in critically auditing the identification of meaning patterns (themes).
**8**. Forming a consensus on themes:	The themes were finally formulated, revised and agreed upon by all four authors.

A “meaning pattern” can be defined as a condensed summary of the units of relevance for a particular research topic that may be identified when comparing the experiences of several participants (Binder, Holgersen, & Moltu, 2012). A pattern is said to emerge when there is a high degree of convergence between the experiences of different participants within a certain area, and at the same time a moderate degree of divergence between them that makes the pattern thematically rich. The meaning patterns were conceptualized by formulating themes, and providing quotes from the material to exemplify the content of these themes and enhance the transparency of this interpretive approach (Binder, Holgersen, & Moltu, 2012). The interviews with the participants differed considerably in terms of the level of detail and thickness of descriptions. This was addressed by analyzing the entire data set, but selecting quotes from six participants with especially rich descriptions to illustrate the overall themes and meaning patterns that in themselves were formulated on the basis of all interviews in the sample. The examples in these six interviews were found to be representative of the thematic content of the 29 interviews analyzed in this study.

### Ethics

The study was approved by the Regional Committee for Medical and Health Research Ethics (REK VEST, project number: 021.04) and the Norwegian Data Protection Authority (reg. code: 87478942). Care was taken to provide adequate information about the study, and written informed consent was obtained from all students prior to participation in the study. The participants were prescreened for potential clinical risk using the exclusion criteria. Students were informed that they could contact teachers during the MBSR program if they experienced emotional distress.

## Results

**Figure 1:**
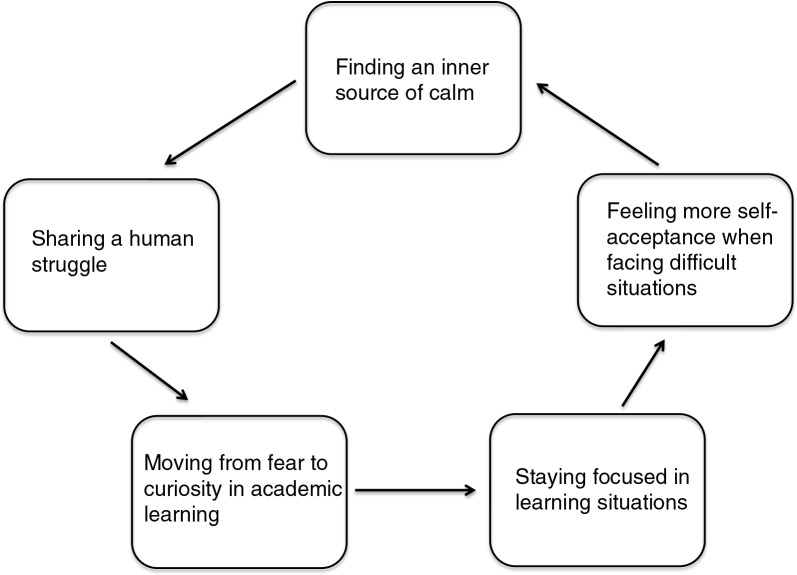
Summary of findings (themes).

Participants’ descriptions of their experiences when taking the MBSR program clustered around five main themes: (1) finding an inner source of calm, (2) sharing a human struggle, (3) staying focused in learning situations, (4) moving from fear to curiosity in academic learning, and (5) feeling more self-acceptance when facing difficult situations (Fig. 1).

### Finding an inner source of calm

An initial theme in our analysis was the participants’ experiences of finding an inner source of calm through the mindfulness meditation exercises. In the pre-course screening interviews, the participants disclosed feeling overwhelmed by worries, physical arousal, and negative feelings before academic deadlines, often to a degree that impaired their ability to study and engage fully in their everyday life. Participants had often struggled with performance anxiety for years, with an accumulated sense of futility, frustration, and fear of failure.

Most of the participants encountered initial difficulties with the meditation practice. They described restlessness, frustration, fatigue, sleepiness, and concentration difficulties during the mindfulness exercises. However, they noted that the daily homework assignments gradually shifted during the 8 weeks of the course from being an extra burden to becoming a welcome relief in their everyday life:

In the beginning it was kind of something you had to do before you could relax. But then towards the end of the exercise it felt good, it could be a positive thing rather than something you had to do before you could go to sleep, like it was in the beginning. At first I did not really understand what I was doing, I just said to myself: “I just have go through this somehow.” But then you notice: I slept better after I started doing the exercises. I slept much calmer, and felt calmer generally as well. Then I found out that it had an effect, and it became much easier to do the exercises. (Female A, 24 years)

The practice of intentionally focusing awareness on their breathing and bodily sensations was experienced by most of the participants as a helpful way to regulate negative mind states such as ruminative thoughts or feelings of fear, frustration, and shame. They described using these meditation practices as useful practical techniques to “step back” from these experiences, and focus on their breathing as they noticed bodily sensations, emotions, thoughts, and their immediate surroundings. Some used this as a technique in specific anxiety-provoking situations, whereas others began to practice these exercises regularly and described feeling a greater sense of calm and emotional balance in their everyday life. Some participants described this learning experience as finding “a source of inner calm” where mindful awareness of breathing had helped them to regulate the intensity of emotional distress and relate to negative feelings from a different perspective. The experience of having practical ways to access feelings of calm provided the participants with a growing sense of agency within the storm and stress of their everyday lives:

And I feel a different relationship to stress. When it begins, when it starts to get too much … too much to focus on, and I feel that stress is starting to build up, I have the exercises as a kind of refuge. So I have a way to bring myself back down to earth and just tell myself that: “It's no big deal, now I can just go and do the exercises, and then it will be fine afterwards.” So I know that it can really change how I feel inside when I do the exercises. (Female B, 47 years)

### Sharing a human struggle

Another important theme was how the experience of encountering other students with the same problems helped the participants to feel less alone with their performance anxiety. Many participants described a long-term struggle with self-criticism and emotional isolation due to their academic difficulties. For example, in the beginning of the program, they expressed doubts if they were “doing it right” or thinking that “there must be something wrong with me” when they were unable to feel relaxed or focused during the exercises. Hearing how other students struggled with the same challenges during the mindfulness exercises normalized these experiences for the participants. One participant described how the process of understanding anxiety reactions as shared existential challenges made it easier for her to continue with her own meditation practice:

It was good to hear that there were several others who had the same problems as me when it came to exams. And at times, when I felt restless or discovered that I had slept through half the body scan exercise, there were others who had done the same. It was not just me who faced those challenges there. And at the same time it felt good to hear when they got it right, because it sort of told me that if I just kept going—when I was feeling stuck, and the others—someone described that they had a great experience the day before … then I thought: “If I just keep going, it can change.” So in that sense it helped me to continue. (Female C, 23 years)

Many participants felt ashamed about their performance anxiety, and described relief in realizing that they were not alone in their suffering. The group discussions provided opportunities to talk openly about potentially embarrassing experiences associated with academic evaluation anxiety:

There was one student who asked why she constantly had to go to the bathroom when she had exams. And it's one of those questions that you usually don't dare to ask, such as: Why does my stomach turn inside out? It's not that you actually just get the medical explanation for why it's like that, but that you can actually talk about it with others. And I think we discussed blood pressure and other things you usually don't see as being related to stress, and it was interesting to hear that there were many common symptoms, even if it doesn't show on the outside. So it was reassuring to know that I'm not the only one who has to run to the bathroom when it's like that (laughs). So you don't feel so alone, you feel more normal. (Female D, 23 years)

The quote above illustrates how the participants could discuss their fears of being different and finding resonance in the experiences of others. After recognizing their shared struggles with academic evaluation anxiety, the participants described being more open to talking about their vulnerable feelings within the group and engaging constructively with their own difficulties between the classes. The participants described this as making them feel more normal and enabling them to recognize anxiety as a natural and workable human struggle.

### Staying focused in learning situations

The participants described that they began exploring ways of using mindfulness when they were distracted by anxious feelings in academic performance situations. The experience of learning to allow anxious thoughts and feelings in the mindfulness exercises provided a model for many participants in terms of how they could approach similar distractions in academic learning situations. Some students actively used the mindfulness practices to prepare for exams and reduce their general stress levels, whereas others used these exercises in moments of “acute” performance anxiety in exam situations:

I have been very troubled by losing my focus during exams, that I can't concentrate on the task and what I do. And it just becomes a snowball rolling downhill and just growing bigger … Finally I get very stressed. But now I use the breath, and focus on my breathing as soon as I enter the room. So when the exam questions are handed out, I'm already quite calm and focused, and don't have the tendency to look up right away to see what is happening around me, or what the others are writing. (Female C, 23 years)

One participant discovered that accepting these distractions would paradoxically improve her capacity for concentration, stating that “if distractions happen, I know that it's not my fault; I just go back and keep coming back again, and again, and again” (Female A, 24 years). The students described turning their attention to their breathing or bodily sensations when encountering negative thoughts and feelings as they were attempting to concentrate on their studies. Participants described using awareness of their breathing when reading academic material, writing a paper at home or writing an essay in an actual exam situation, performing in a musical recital, or when doing presentations in front of others. The mindfulness exercises helped them to be more present when they were studying or taking difficult exams: “Those situations that could previously be stressful were now more … I was able to concentrate and focus on exactly where I was, and what I was doing” (Female D, 23 years).

### Moving from fear to curiosity in academic learning

An important theme in our analysis was how some of the participants began exploring new ways to approach the process of academic learning. Many participants described that their learning strategies and perceptions of their studies were strongly influenced by fear and avoidance behavior, such as actively avoiding study activities, worrying about low grades or ruminating about academic failure. Some of the participants described examining their personal ways of reacting to academic stress during the course, and began experimenting with a different stance toward their own learning process. These participants described a gradual shift in their learning situation as a whole, where they experienced more curiosity in, and discovery from their own academic learning process. One participant, a music performance student, described a subtle but important change in how she would approach her own performance at music recitals:

I can somehow separate who I am from the result of my performance. It's like I am the one that breathes—and at the same time I am also the one who does something else. But I'm sort of two things at the same time. (Female A, 24 years)

Another participant described a movement from fear of failure to feeling motivated by interest and curiosity in her academic studies:

I worked in a different way. I worked much more focused, and much calmer. And I always knew what I wanted to do, and did not use a lot of time wondering: What is the right thing to concentrate on now, what should be the next step? It came completely by itself, for I was not thinking about how anyone would evaluate me, I sat and thought about my own process and my own learning and focused on what I wanted to do with the material I had in front of me. So it was different. I think I have become more self-driven, I would say that I have for many years been very externally driven—probably for all my life and it's probably a lot of what has made me stressed and very afraid of what others will think of me. But I think I might have become a little less scared now. I was preparing the exam mostly for myself, and not for the examiner or anyone else for that matter. It felt good. (Female B, 47 years)

The same participant described that she concentrated more on understanding the “bigger picture” and the unifying “threads” of the academic subject:

I think I've spent a lot of my capacity on being stressed, dreading, having catastrophic thoughts and worrying about what lies ahead of me. And it has restricted me a lot in terms of how I have dealt with the academic material. It sort of becomes small puzzle pieces, an awful lot of small puzzle pieces that in some way are supposed to become a meaningful picture in your head. But this time it hasn't been like that, because the big picture and the red thread have been there. (Female B, 47 years)

The quote above illustrates an important shift from “catastrophic fears” and “getting lost in the details” toward seeing the learning process itself from a more balanced meta-perspective. Some participants would find themselves becoming more oriented toward the learning process itself and their own long-term mastery goals, and worrying less about immediate exam results or the possible reactions of others. This approach to academic learning appeared to allow them to enjoy the prospect of gaining new insights and gradual mastery in their academic field, even when facing stressful examination periods.

### Feeling more self-acceptance when facing difficult situations

A final theme in our analysis was the participants’ experiences of feeling more self-acceptance in difficult situations. When describing their presenting problems, the participants disclosed strong internal images of how they “should” or “must” be. After the course, many of the participants described being more accepting toward themselves at times when they were struggling in daily life. They described having more inner space to allow negative feelings or unpleasant sensations to come and go, being more aware of the shifting nature of negative mind states, as well as having a more skeptical attitude toward self-critical and catastrophic thoughts. One of the participants described her experience of feeling more self-acceptance in these situations:

I have become more balanced and less afraid to tackle unexpected situations. Thinking more like “yes, things are not …” I don't take things too seriously. To accept that it doesn't always work out as you wish and, simply, be less critical towards yourself. I do have a tendency to take on the blame for things, but now I really just think that there it's not my fault, in a way. That's not my fault. And just feel more self-acceptance, of some sort. (Female E, 24 years)

The experience of feeling self-acceptance in these situations was rarely described as a linear process, or a global and permanent achievement. Sometimes the participants described falling back into familiar patterns of self-condemnation, doubt and insecurity, but that they were gradually able to be kinder toward themselves in difficult situations. Several participants described that they could still feel anxiety after the program, but that they had learned to accept these reactions and relate to them in a new way:

I may perhaps have the same nervousness, yet I can relate to it in a different way. It's still there, but I don't get so caught up or paralyzed, I can sort of allow to let it be. I remember we talked in the course about letting things be the way they are, instead of constantly trying to escape things that are uncomfortable. So I do feel I have learned something, it's easier for me to relate to my own nervousness. (Female F, 48 years)

Having learned practical strategies such as awareness of breathing as a way to accept and be present with anxious feelings, participants described that they could allow their anxiety to be there and still study or perform in exams. Participants experienced being more supportive of themselves or even laughing quietly to themselves about their reactions in difficult situations. Some participants described being less afraid of their own feelings and more attentive to feelings and the need for self-care during stressful exam periods. One participant described that this experience of being less afraid of anxious feelings made it easier for her to face challenging performance situations:

I think I look a little more positively at things now. A bit like this: “Well, I am nervous, ok, yes, interesting, yes, fine, fine.” Then I go on, rather than making it into an obstacle. And before the exam, I feel it helps to just sit down for ten minutes, to get some perspective, get some sort of overview, I think it makes me a bit calmer and in control, and … feeling that the nervousness is there, but I can breathe at the same time. It may still be here, but you don't drown in the nervousness. (Female A, 24 years)

## Discussion

We have presented our analyses of the experiences of the 29 participants, focusing on five themes identified in the accounts offered by the participants in our study. Figure 1 provides a visual summary of our themes.

The themes identified in our analysis indicate something of the range and complexity of the participants’ subjective experiences during the 8-week MBSR course. When taken together, the themes developed from our analysis indicate that the group-based mindfulness intervention seemed to offer the participants more than just help with their specific presenting problem of academic evaluation anxiety. The participants described experiencing meaningful forms of change in different domains of their everyday life, and we will discuss and contextualize these findings in terms of three dimensions: (1) The process of developing capacities for inner calm and self-acceptance, (2) the experienced impact of group processes in promoting self-acceptance, and (3) the experience of learning mindfulness to master difficulties in academic contexts.

### Understanding the process of developing capacities for inner calm and self-acceptance

The themes of “finding an inner source of calm” and “feeling more self-acceptance when facing difficult situations” are different, but at the same time closely related experiences that describe the broader process of exploring new ways to face emotional distress and personal suffering. The experience of being able to calm oneself (theme 1) seemed to offer new possibilities for both agency and acceptance in the face of anxiety. The sense of practical mastery or coping ability that the exercises offered the participants appeared to promote an increased sense of agency when encountering stress and anxiety in everyday life. The participants’ descriptions of using the mindfulness exercises as practical techniques to regulate negative feelings (theme 1) may resonate with research which indicates that mindfulness may enhance capacities for emotional regulation (Chambers, Gullone, & Allen, 2009). Robins, Keng, Ekblad, & Brantley (2012) found that the MBSR program might have a beneficial impact on clinically relevant emotion regulation processes. Our findings do not provide causal explanations for the experiences described by the participants, but may possibly offer a phenomenological or first-person description of these forms of emotional and attentional regulation experienced and felt at the level of the participants’ subjective experience. The program also appeared to have an impact on how the participants experienced and acted toward themselves in difficult situations. The participants’ experience of finding self-acceptance in difficult situations (theme 5) seems to resonate with theoretical formulations of an accepting or compassionate stance toward the self as an important dimension of change in psychotherapy (Gilbert, 2010; Rogers, 1995; Williams & Lynn, 2010). Neff and McGehee (2010, p. 226) describe self-compassion as “the ability to hold one's feelings of suffering with a sense of warmth, connection, and concern” and argues that this attitude of acceptance when facing pain or failure represents an important pathway to mental health. Increased self-acceptance and a greater capacity for emotional regulation (inner calm) have been reported as recurrent findings in qualitative studies on client experiences in mindfulness-based interventions (Malpass et al., 2012; Wyatt et al., 2014). For example, Allen, Bromley, Kuyken, and Sonnenberg (2009) conducted a qualitative study of mindfulness-based cognitive therapy for recurrent depression and identified “acceptance” as an important theme in the clients’ descriptions of their experience. They described the theme of acceptance as involving both a new way to understand the self (destigmatization) and a new way of relating to depressive thoughts and feelings (depression objectified). The possibility of cultivating a compassionate stance toward the self through meditative practice has been a central concern in the mindfulness literature (Germer, 2012; Gilbert, 2010), and studies indicates that group-based mindfulness-based interventions may promote increased levels of self-compassion (Baer, 2010a; Kuyken et al., 2010; Neff & Germer, 2013; Shapiro, Astin, Bishop, & Cordova, 2005). A potential benefit from our study is the first-person perspective on how participants in a mindfulness-based group intervention experience the process of developing self-acceptance or a compassionate stance toward the self. Our findings did not suggest an immediate transformation in the global self-concept of the participants we interviewed, but seemed to indicate gradual changes in their experience of being more accepting toward themselves in specific contexts.

### The role of group processes in mindfulness-based interventions

The experience of “sharing a human struggle” (theme 2) is also a theme that strongly relates to the experience of increased self-acceptance, and that seems most closely tied to more generic group processes within the MBSR program. Acknowledging anxiety as a part of the human condition could be an important way to counteract the isolation and shame experienced by the participants in their struggles with performance anxiety. An important question in understanding these findings is to what extent these experiences are specifically related to the mindfulness interventions in the MBSR program, and to what extent they represent processes and principles found in other psychotherapeutic approaches. The meditation practices may represent a specific factor in the MBSR program, but the finding that the group experience itself appeared to reduce feelings of isolation among the participants indicates the potential role of group processes and common factors in mindfulness-based interventions. The psycho-educational components and group structure of the MBSR program emphasize suffering as a normal and universal feature of the human condition. Imel, Baldwin, Bonus, and MacCoon (2008) examined results from 59 MBSR groups and reported group membership as accounting for 7% of the global variability in psychological outcomes, which indicates the potential significance of group dynamics in MBSR. The experience of a supportive group environment has been reported in prior qualitative studies as an important factor that may enhance or facilitate change in mindfulness-based interventions (Malpass et al., 2012; Wyatt et al., 2014). MacKenzie, Carlson, Munoz, and Speca (2007) conducted a qualitative study of MBSR for cancer patients and described the significance of “shared experiences” and a supportive group environment as an important theme in the narratives of the patients in their study. The importance of group cohesiveness and social support have been described as important psychological processes in the group therapy literature (Yalom & Leszcz, 2005), but have not yet become a major focus in empirical research on change processes in mindfulness-based group interventions (McCown, Reibel, & Micozzi, 2010).

### Learning mindfulness as a way to master academic problems

The themes of “staying focused in learning situations” and “moving from fear to curiosity in academic learning” might represent experiences specific to students in academic contexts, or may describe more general processes involving a movement from being driven by avoidance to developing mastery in relation to anxiety symptoms and problems in life. Although there are no prior empirical studies that have examined mindfulness-based interventions for performance anxiety in university contexts, the participants’ descriptions of their experiences of learning mindfulness as a way to master academic problems have points of convergence with research in cognitive psychology, neuroscience, and academic learning. The participants’ experiences of being more able to stay focused in learning situations (theme 3) may have important parallels with cognitive models of test anxiety and neuroscientific research on mindfulness. Attentional control theory (Derakshan & Eysenck, 2009) postulates that anxiety interferes with the cognitive resources available to process complex tasks in performance situations. Neuroimaging studies indicate that systematic training in mindfulness meditation might produce improved concentration and attentional processes (Chiesa, Calati, & Serretti, 2011). Farb et al. (2007) conducted a neuroimaging study of participants in an MBSR course, and found evidence of neural dissociation between two different forms of self-awareness: “narrative focus,” which involves monitoring of self-referential traits, and “experiential focus,” which entails broadly attending moment-by-moment experiences. These two forms of self-awareness seem to involve distinct neurobiological systems. Farb et al. (2007) argue that the MBSR program may decrease rumination and self-referential processes by developing capacities for focused attention to the present. The participants’ exploration of new approaches to academic learning may provide an experiential perspective on processes described in prior research on motivational processes in learning. The participants’ descriptions of moving from fear to curiosity in their academic learning (theme 4) can be seen as resonating with Dweck's (1986) descriptions of learning versus performance goals, and Elliot's (1999) distinction between approach and avoidance motivation in understanding achievement goals. Dweck (1986) differentiates between performance goals, where individuals attempt to gain favorable evaluations and avoid negative judgments of their competence, and learning goals, where individuals attempt to increase their competence to understand and master new areas. Whereas performance goals involve demonstrating competence relative to others, learning goals are driven by intrinsic motivation and the individual's development of mastery. Elliot (1999) describes avoidance motivation as behavior directed toward a negative or aversive possibility, whereas approach motivation is described as behavior that is directed toward a positive event. Neff, Hsieh, and Dejitterat (2005) found that self-compassion among undergraduate students was positively associated with adaptive academic motivation patterns (learning goals) and negatively associated with performance-avoidance goals, indicating that self-compassionate students might cope more adaptively with the fear of academic failure. The participants’ descriptions of their performance anxiety and prior efforts to avoid academic failure may illustrate such maladaptive attempts to master the process of academic learning. The themes of staying focused when facing distractions (theme 3) and moving from fear to curiosity in academic learning (theme 4) may illustrate how moving toward a growth and mastery-oriented approach may be helpful for university students struggling with performance anxiety.

Most of the participants described that they experienced the course as relevant and useful for finding ways to master their academic evaluation anxiety, however many also described that the 8-week course provided them with more help than for their presenting problem of academic evaluation anxiety. The qualitative meta-syntheses conducted by Malpass et al. (2012) and Wyatt et al. (2014) indicate a similar multidimensionality of the experiences and change processes described by clients participating in other studies of mindfulness-based interventions. The MBSR program emphasizes the interplay of formal mindfulness practices (the body scan, sitting, standing, and walking meditation exercises) and informal mindfulness practice in ordinary everyday situations, with the assumption that the effects of these formal exercises might generalize into other domains of daily life. Many participants described that the program would not only have an impact on their experience of performance anxiety in academic contexts, but would influence how they felt about themselves and engaged with difficulties in other situations in their everyday life. The wide range and complexity in the client experiences identified in this particular study suggest that a qualitative investigation of the subjective experience of participants may be important to understand the contextual factors and multiple dimensions of change in mindfulness-based interventions.

### Reflexivity

Reflexivity involves continuously examining how the subjectivity and preconceptions of the researcher or team of researchers may affect the understanding and interpretation of the phenomena in the research process (Alveson & Sköldberg, 2009; Binder, Holgersen, & Moltu, 2012; Finlay & Gough, 2003). The contemporary popularity of mindfulness-based interventions calls for a reflexive recognition that these interventions, like other psychotherapeutic approaches, may not work for everyone. Our primary interest when developing the interview guide for this study was to explore the perceived relevance and usefulness experienced by university students undergoing the mindfulness-based intervention, and to understand how participants with academic evaluation anxiety experience would integrate the 8-week program into their everyday life. The fact that three of the researchers in our team have practiced mindfulness and taken an MBSR program might offer both advantages and disadvantages. Having a personal background as mindfulness practitioners gives the advantage of first-hand knowledge of possible client experiences that could be explored in the research interviews with the participants, but may also have constricted our openness for recognizing other important processes among the participants in the MBSR program. Researchers without a personal mindfulness background might identify different phenomena during the research process, and the presence of a “non-practicing” researcher in our research team represented an important part of the critical auditing in our research process. For example, the critical examiner in the research team highlighted important relationships between the five themes we identified in this particular MBSR study, and identified an important “meta-theme” or narrative structure in the themes we identified, where several of the individual themes described the participants’ experiences as initially encountering a negative experience (“challenge”) and then having a positive experience (“mastery”). The positive trend in this meta-theme may represent an accurate interpretation of the experiences described by the participants in the qualitative interviews, but may also highlight the need for discussing the limitations of this qualitative study.

Another important question that emerged in the reflexive process was the possibility of gender differences in academic performance anxiety. The participants who entered our study were predominately female university students (86% women vs. 14% men), and only 4 among the 29 students we interviewed were male. The low participation of male students in this study may reflect gender differences in coping strategies when faced with academic stress and performance anxiety; or that male students were more reluctant to seek professional help.

### Limitations of the study

There are several important limitations in the present study. The explorative methodology used in this article enables a richer in-depth exploration of the participants’ experiences during the MBSR program. This explorative qualitative design does not aim to answer questions of how frequent these experiences are in mindfulness-based interventions, or the possible causal mechanisms that may explain these experiences. In other words, qualitative studies cannot demonstrate that MBSR had causal effects on performance anxiety or academic performance.

The possibility of sample characteristics also limits the generalizability of our results. We recruited participants with performance anxiety to our study through advertisements and self-referrals and cannot rule out that these self-selected students were different from the larger student population. The majority of participants interviewed were young students (70% were under 26 years of age), but the sample also included five participants over 35 years of age, which raises the question of how their age differences and personal characteristics might have influenced the interview data in our study. Furthermore, the sample only included a very limited number of male students. The six participants we used to provide the “rich quotes” to illustrate the findings were all female, which may represent an important question to the present study. We cannot, from the findings in this study, assume that the MBSR program is equally accessible and acceptable to both male and female university students.

### Implications for research

Qualitative research methods have the potential of systematically exploring user experiences and subjective processes of change in mindfulness-based interventions such as MBSR (Castonguay & Beutler, 2006; Elliott, 2010; McLeod, 2011), and our findings do indicate what clients may experience as useful and relevant when participating in mindfulness-based interventions for anxiety problems. It may be important to secure a balanced gender sample in future studies to assess the impact and implications of possible gender differences among university students with academic performance anxiety. Further empirical studies will be required to investigate the causal mechanisms of change in mindfulness-based interventions for academic evaluation anxiety. We recommend that future studies investigate both levels of performance anxiety, subsequent academic performance and general mental health as possible outcomes of psychological interventions for this population.

## Conclusion

In this study of 29 university students with a history of academic evaluation anxiety, we found the following themes in their reported experiences of the MBSR program: (1) providing them with ways to find an inner source of calm when experiencing stress and anxiety, (2) that the group experience itself was described as reducing feelings of shame and increasing understanding of anxiety as a shared human struggle, (3) that students applied these exercises to stay focused in different learning and performance situations, (4) that some students would explore new approaches to academic learning characterized by curiosity and mastery orientation rather than fear of failure, and (5) that participants described feeling more self-acceptance when facing difficult situations. We have discussed and contextualized our findings in relation to existing research, discussed our process of reflexivity, as well as highlighted important limitations and possible implications for future research.

## Appendix

**Interview guide**

**Introduction**

Can you tell me a bit about what made you sign up for this course?

Possible follow-up questions/probes:

- What were your symptoms?
- How long did your symptoms last?
- What made you want to seek help?
- What were your expectations?

**Experiences during the MBSR course**

Can you tell me how it has been for you to attend the course—from the beginning to the end?

General follow-up:

- What has been useful for you in the course?
- What has been negative/less good/challenging/uncomfortable for you?

Follow-up on group experience:

- How have you experienced doing the exercises in the group?
- How has it been for you to share experiences with the others in the group?
- How has it been for you to hear others talk about their experiences?

Follow-up on the mindfulness exercises:

- How have you experienced the exercises you have been introduced to?
- How has it been for you to do exercises at home? (hindrances and solutions)
- Which exercises were most helpful for you?
- Which exercises were least helpful for you?
- How has it been for you to:
  - focus on bodily sensations (body scan, yoga)?
  - focus on the breath?
  - focus on the thoughts/feelings?
  - be aware of everyday life?
- Are you going to continue to do some of these exercises? If so, which ones?
- When it is difficult to pay attention or to do exercises are there other things that help you?

**Changes after the MBSR course**

Have you experienced that something in your life has changed during or after the course?

Possible follow-up questions:

- Are you doing something different today—can you possibly describe an episode where you did something different than before?
- Do you experience any changes in how you cope or deal with your difficulties? Ask more specific about coping with:
  - stress
  - anxiety
  - ruminations
  - depressed mood/depression
- Has learning mindfulness affected your relationships with other people? For example, with regard to:
  - listening to or paying attention to what others have to say?
  - noticing what happens to you when you are with others?
  - what you choose to do or how you behave when you are with others?
- If you plan to continue with the exercises do you think that there might be some specific changes in the long term?

**Conclusion**

- Are there other things I have not asked you about but which would be important to include?
